# Self‐Metalation of Porphyrins at the Solid–Gas Interface

**DOI:** 10.1002/anie.202111932

**Published:** 2021-11-09

**Authors:** Francesco Armillotta, Enrico D'Incecco, Manuel Corva, Matus Stredansky, Jean‐Jacques Gallet, Fabrice Bournel, Andrea Goldoni, Alberto Morgante, Erik Vesselli, Alberto Verdini

**Affiliations:** ^1^ Physics Department University of Trieste via Valerio 2 34127 Trieste Italy; ^2^ CNR-IOM, Area Science Park S.S. 14 km 163.5 34149 Basovizza Trieste Italy; ^3^ Sorbonne Université CNRS UMR7614 Laboratoire de Chimie Physique Matière et Rayonnement 4 place Jussieu 75005 Paris France; ^4^ Synchrotron SOLEIL L'Orme des Merisiers, Saint-Aubin—BP 4891192 Gif-sur-Yvette CEDEX France; ^5^ Elettra Sincrotrone Trieste S.S. 14 km 163.5 34149 Basovizza Trieste Italy

**Keywords:** near-ambient pressure, porphyrins, self-metalation, sitting-atop complex, tetrapyrroles

## Abstract

Self‐metalation is a promising route to include a single metal atom in a tetrapyrrolic macrocycle in organic frameworks supported by metal surfaces. The molecule–surface interaction may provide the charge transfer and the geometric distortion of the molecular plane necessary for metal inclusion. However, at a metal surface the presence of an activation barrier can represent an obstacle that cannot be compensated by a higher substrate temperature without affecting the layer integrity. The formation of the intermediate state can be facilitated in some cases by oxygen pre‐adsorption at the supporting metal surface, like in the case of 2H‐TPP/Pd(100). In such cases, the activation barrier can be overcome by mild annealing, yielding the formation of desorbing products and of the metalated tetrapyrrole. We show here that the self‐metalation of 2H‐TPP at the Pd(100) surface can be promoted already at room temperature by the presence of an oxygen gas phase at close‐to‐ambient conditions via an Eley–Rideal mechanism.

## Graphical abstract

The catalytic self‐metalation of tetrapyrroles at surfaces can be promoted by oxygen at the solid–gas interface already at room temperature, lowering the reaction barrier as in the case of 2H‐TPP/Pd(100).

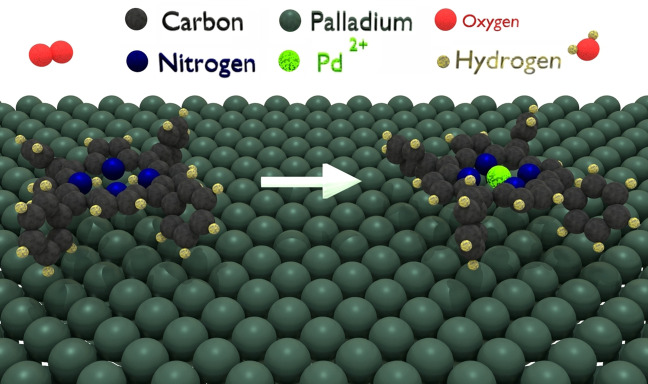

## Introduction

Porphyrin metalation occurs in Nature thanks to the catalytic mediation of enzymes. The specific insertion of a metal ion into tetrapyrrole macrocycles is catalyzed by a group of enzymes called chelatases.^[1]^ The main step of the metalation mechanism consists of a distortion of the tetrapyrrole occurring at the anchoring pocket site of the catalytic protein.[^1^, ^2^, ^3^] Within this picture, the enzyme fixes the pyrrole rings and induces a tilt in one of them, allowing the metal to enter the porphyrin.^[2]^ A fundamental role is played by the active site residues, as in the case of human ferrochelatase, that actually control the stereospecific distortion for metal insertion.[^1^, ^3^] From the surface science point of view, adsorption of tetrapyrroles at surfaces provides a suitable environment for metalation, yielding both necessary charge transfer and geometric distortion.[^4^, ^5^, ^6^] In particular, self‐metalation is a promising route, where the metal inclusion proceeds by simply picking up a surface atom.^[7]^ However, within a surface science approach based on model systems in ultra‐high vacuum (UHV) conditions, often an activation barrier needs to be overcome, which can be even higher than 1 eV, depending on the molecule, the surface, and the metal.^[8]^ In selected cases, like for the self‐metalation of 2H‐TPP/MgO(001), pre‐oxidation of the supporting termination lowers the activation barrier of the process thanks to a charge transfer mechanism.^[9]^ On purely metallic surfaces however, although heating the system above 300 K might provide sufficient energy to overcome the barriers, high temperatures might yield permanent damage to the organic framework in selected cases. To tackle this issue, we show here that self‐metalation can be promoted at room temperature at the solid–gas interface by the direct interaction of the tetrapyrrole macrocycle with an oxygen gas phase at near ambient pressure (NAP) conditions. Whereas recent studies have definitely proven the role in UHV of adsorbed atomic oxygen in promoting the metalation reaction via the formation of a sitting‐atop complex and through the production of molecular hydrogen or water,[^8^, ^10^, ^11^, ^12^, ^13^] the extension of this level of insight up to NAP conditions is unprecedented yet.^[14]^ The potential role of an oxygen atmosphere has been addressed in the porphyrin self‐metalation mechanisms observed on Co and Ag terminations in UHV.[^15^, ^16^] However, the concomitant oxidation of the surface makes it hard to distinguish between the role of a direct (Eley–Rideal like) mechanism induced by gaseous O_2_ and a surface‐mediated process. At variance with the mentioned systems, on palladium we find that no surface oxidation takes place under the considered conditions, since the porphyrin monolayer prevents the evolution of a sizeable oxygen surface coverage, and specific insights about the role of gaseous O2 can be thus achieved.

## Results and Discussion

**Figure 1:**
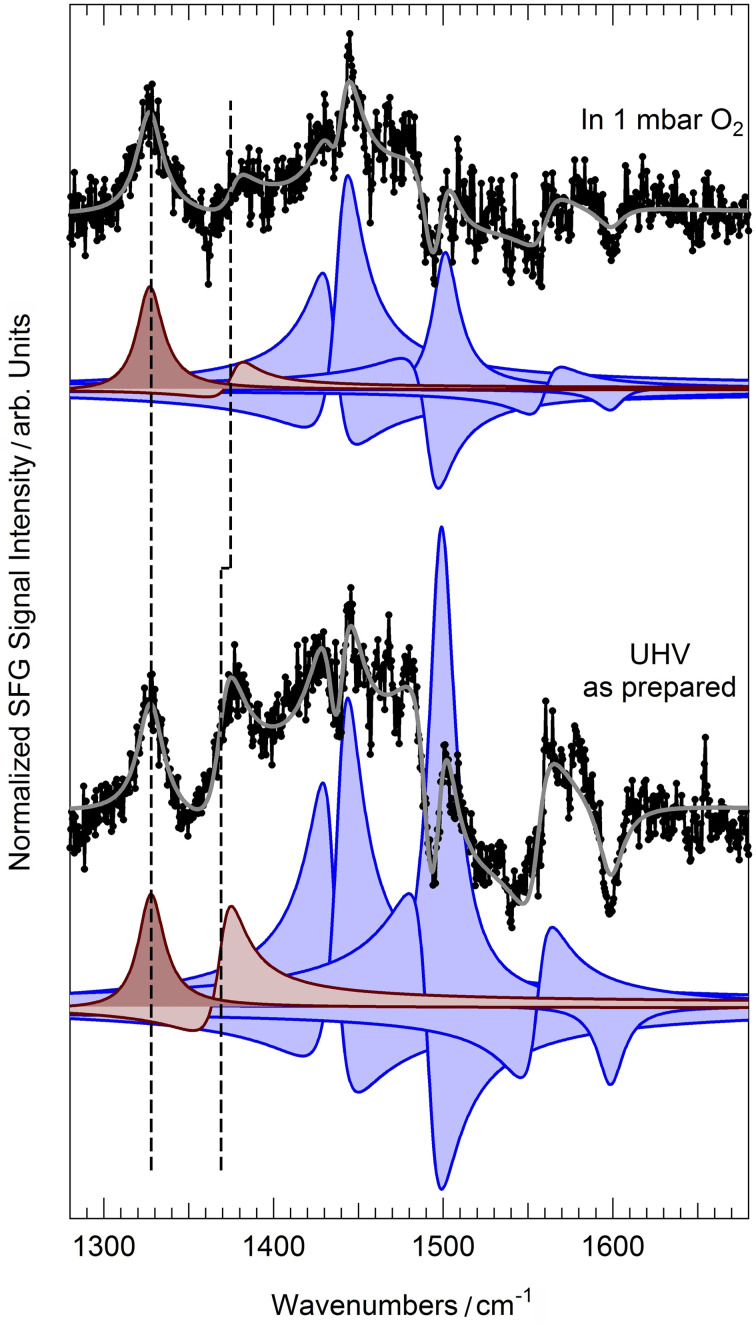
IR–Vis SFG vibronic spectra of (bottom) the 2H‐TPP/Pd(100) monolayer in UHV, as prepared after deposition and annealing to 410 K for best ordering, and (top) in 1 mbar O_2_. Normalized data (black dots) are shown together with the best fit (gray line), obtained according to the line shape described in the text. The deconvolution of the resonances (color‐filled profiles) is also shown. The dashed lines indicate the position of the resonances (brownish) affected by the metalation process. Both spectra were collected at room temperature and with ppp polarization configuration.

**Figure 2:**
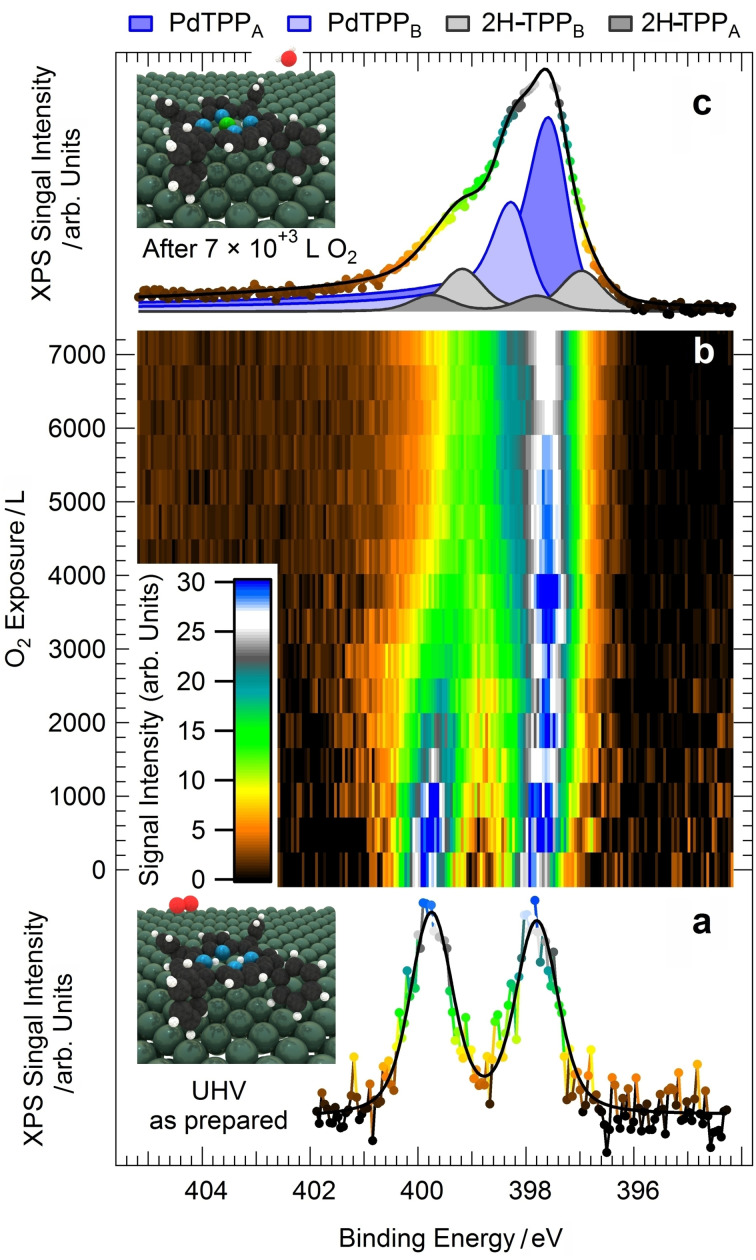
N 1s XPS core level spectra: a) 2H‐TPP/Pd(100) monolayer in UHV, as prepared; b) color map depicting the evolution of the N 1s core level spectra collected in situ as a function of the oxygen exposure; c) N 1s spectrum collected in UHV at the end of the process (all spectra were measured at room temperature and with *hν*=650 eV). 3D models of both 2H‐TPP and PdTPP are shown, together with gas‐phase reactants (O_2_) and products (H_2_O).

**Figure 3:**
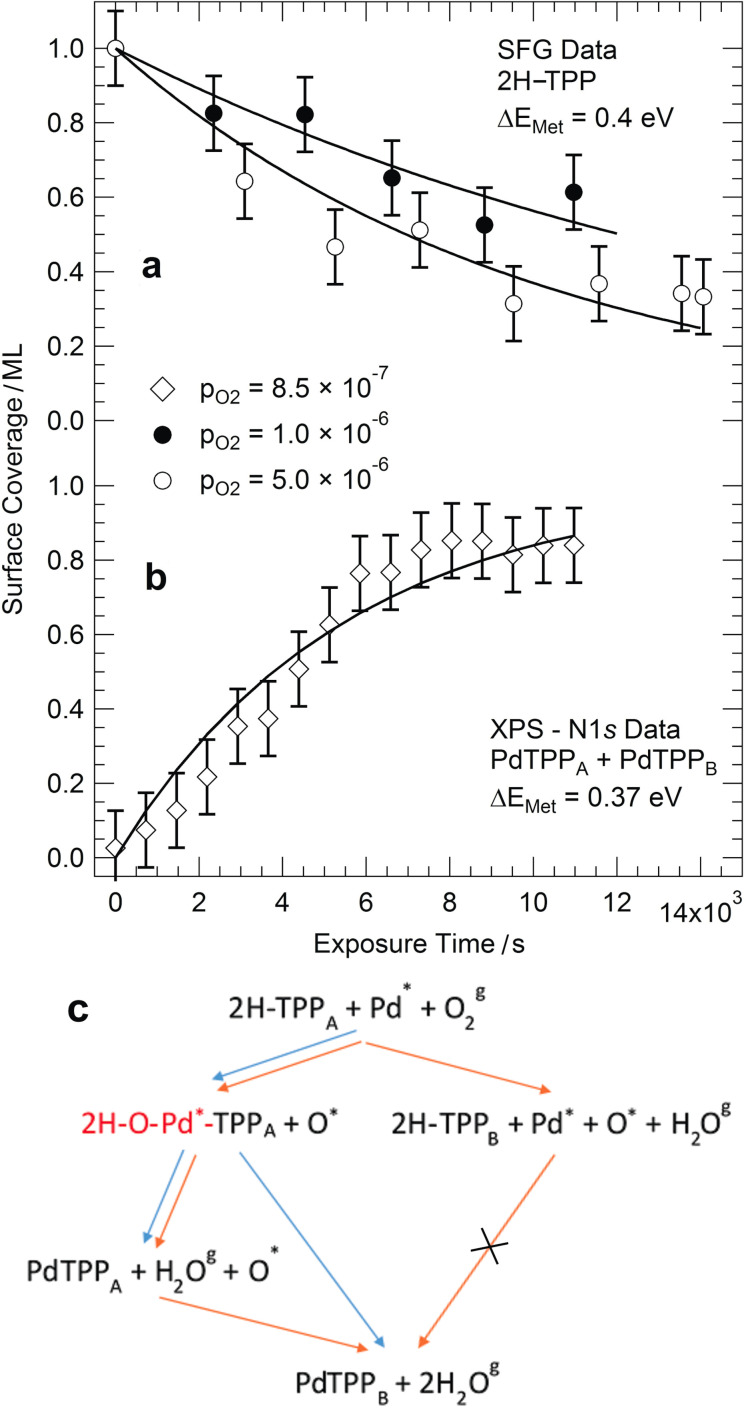
a,b) Coverage evolution of the 2H‐TPP and PdTPP species as obtained from the experiments performed in situ at room temperature during the oxidation process; a) 2H‐TPP coverage as from the IR–Vis SFG data (two independent experiments are reported, performed at 1.0×10^−6^ and 5.0×10^−6^ mbar O_2_, respectively); b) PdTPP coverage obtained from the N 1s core level spectra collected in 8.5×10^−7^ mbar O_2_; continuous lines represent the best fit with a single degree of freedom, according to the kinetic model described in the text, yielding compatible activation barriers of 0.4 and 0.37 eV for the metalation process, respectively; c) proposed reaction scheme, including oxygen‐induced self‐metalation and dehydrogenation processes, as implemented in the first (blue) and second (orange) kinetic models (see text for further details).

**Figure 4:**
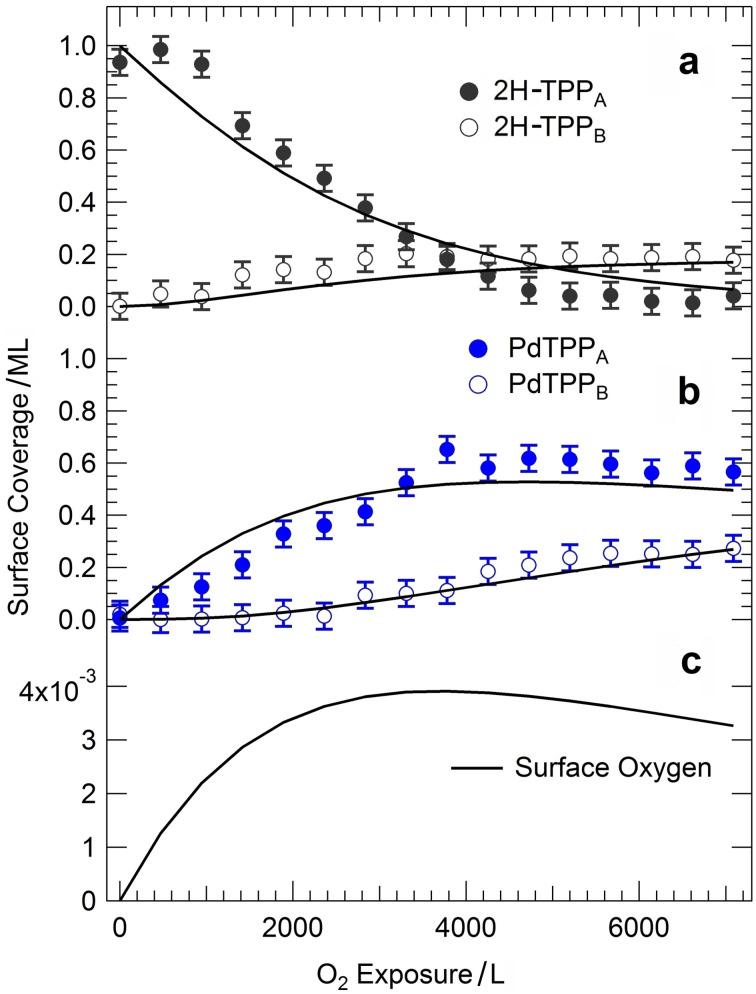
Coverage evolution of a) 2H‐TPP_A_ and 2H‐TPP_B_ species, of b) PdTPP_A_ and PdTPP_B_ species, and c) of surface atomic oxygen O/Pd(100). The experimental data (markers) are obtained from the deconvolution of the N 1s core level spectra collected in situ upon oxidation of the system at room temperature, while continuous lines represent the best fit according to the kinetic model described in the text.

An ordered monolayer of tetraphenyl porphyrins (2H‐TPP) can be prepared on Pd(100) by deposition of the molecules on the substrate at room temperature, followed by annealing in UHV to 410 K to induce best ordering. In the bottom panel of Figure 1 we report the IR–Vis SFG spectrum of the as‐prepared 2H‐TPP monolayer, collected at room temperature in UHV conditions. As recently observed,^[8]^ eight vibronic features can be resolved in this low energy spectral region (filled profiles). Distinct resonances are observed at 1327, 1369, 1436, 1442, 1492, 1499, 1558, and 1600 cm^−1^ accounting for several stretching, bending, and rocking modes.[^8^, ^17^] Annealing of the organic layer in UHV up to 470 K (Figure S1) does not induce significant modifications in the vibronic spectra, thus indicating good stability and poor reactivity of the molecules. Accordingly, it is already known that the system does not self‐metalate upon annealing in UHV.^[8]^ For the present purposes, we will concentrate our attention on the two lowest energy features of the bottom spectrum in Figure 1 (brownish filled profiles). The intensity ratio of the resonances at 1327 and 1369 cm^−1^ can be exploited as a metalation fingerprint when the 2H‐TPP monolayer is deposited on the oxygen pre‐covered Pd(100) termination and subsequently annealed to 410 K, thus inducing metalation and water production at variance with the 2H‐TPP/Pd(100) case.^[8]^ In analogy to the cobalt substrates in vacuo,[^10^, ^15^, ^18^] we exploit here the same metalation marker to extend the investigation beyond the UHV limits, thanks to the unique selection rules of the sum‐frequency generation process.[^19^, ^20^] In the top part of Figure 1 we plot the IR–Vis SFG spectrum of the as‐prepared system (bottom), at room temperature, but in presence of 1 mbar O_2_. Already at a first glance an overall, general diminution of the amplitudes is evident. Moreover, the feature at 1369 cm^−1^ undergoes a blue shift of 6 cm^−1^ and almost disappears, yielding a lower relative amplitude with respect to the resonance at 1327 cm^−1^. Based on literature data,[^8^, ^17^] we observe that three modes actively contribute to the former resonance: the C_β_−C_α_−NH asymmetric stretching, the H rocking of the macrocycle pyrrolic moieties, and the C_α_−N−C_α′_ stretching. On the contrary, the lowest energy feature mainly originates from the phenyl H rocking modes. The spectroscopic information suggests therefore a strong local deformation of the C−N bonds and the loss of the central H atoms, compatible with the metalation to PdTPP molecules.[^8^, ^10^, ^18^] Thus, we clearly see that self‐metalation is induced by the oxygen gas phase at near‐ambient pressure already at room temperature. To confirm the picture and with the aim of obtaining quantitative information about the activation energy of the process, we performed X‐ray photoelectron spectroscopy (XPS) measurements with particular insight into the N 1s core level spectral contributions. The N 1s spectrum of the as‐prepared system (corresponding to the vibronic spectrum in the bottom part of Figure 1) is shown in Figure 2 a. Two symmetric components of equal intensity can be clearly resolved at binding energies of 399.8 and 397.8 eV, associated with the pyrrolic (N−H) and iminic N, respectively,^[8]^ of 2H‐TPPs adsorbed at the Pd surface. Room temperature exposure of the system to oxygen at near‐ambient pressures or, alternatively, prolonged exposure to UHV‐compatible O_2_ background (10^+3–^10^+4^ Langmuir, XPS map in Figure 2 b) yield the N 1s spectrum shown in Figure 2 c. A new peak doublet and two additional, intense singlet features grow at the expense of the initial peaks, as evidenced by the deconvolution (filled profiles) obtained by least square fitting of the data according to the line shapes described in the Supporting Information. In detail, upon oxygen exposure, in addition to the pristine porphyrin species (2H‐TPP_A_—dark gray), a new 2H‐TPP_B_ species (light gray) can be spectroscopically distinguished, associated with pyrrolic and iminic N 1s components at 399.2 and 397.0 eV, respectively. The intense, asymmetric peaks (*α*=0.2, dark and light blue) are associated instead with nonequivalent metalated forms of the porphyrin,^[8]^ and grow at 397.5 eV (PdTPP_A_—dark blue) and at 398.2 eV (PdTPP_B_—light blue), respectively. The intensity evolution of the sum of the two latter peaks is depicted in Figure 3 b, representing the growing surface coverage of PdTPP_A_+PdTPP_B_ as a function of oxygen exposure, at the expense of the unmetalated porphyrins coverage. This information is complementary with respect to the IR–Vis SFG data shown in the upper panel (Figure 3 a), where the monolayer normalized amplitude of the resonance associated with the progressive dehydrogenation and deformation of the macrocycle is reported. The continuous black lines represent the best fitting curves obtained by implementing an extremely simple kinetic model (kinetic model 1, see Supporting Information) with the aim of extracting a quantitative evaluation of the activation barrier for the self‐metalation process. The experimental spectroscopic information provides specific evidence for the evolution of the surface concentration of 2H‐TPP_A_, 2H‐TPP_B_, PdTPP_A_, and PdTPP_B_ species. In order to depict the reaction model, we make the reasonable assumption that O_2_ molecules impinging from the gas phase (Knudsen term) have a reaction/dissociation probability that is proportional to the number of still unmetalated molecules and to the number of free available sites for the accommodation of O atoms at the Pd(100) surface. The full reaction pathway is extremely complex and cannot be accessed with single‐atom level detail since many steps and routes may contribute.^[21]^ A scheme of the overall reaction path proposed in this model is proposed in Figure 3 c (blue path), while the details are reported in the Supporting Information. Within the picture of this model, the O_2_ molecule undergoes dissociation at the center of the tetrapyrroles, contributing at the same time to the formation of the metalation transition state (first O atom, sitting‐atop complex, marked in red in Figure 3 c) and to the oxidation (second O atom, O^*^) of the Pd surface. The sticking coefficient s_0_ is described by a Boltzmann factor, thus including the rate‐limiting barrier Δ*E*_1_ for the process within an Eley–Rideal picture in which metalation is directly induced by the oxygen molecules impinging from the gas phase and dissociating at the tetrapyrrolic sites. We can exclude that the mechanism is a Langmuir–Hinshelwood process since in the latter case O_2_ should first dissociate at the Pd surface yielding O ad‐atoms that then diffuse and induce metalation of the porphyrins. Thus, self‐metalation should occur at room temperature also if oxygen is pre‐dosed at the Pd surface, followed by molecular deposition and this is not the case. Indeed, high temperature is needed to induce self‐metalation on the O/Pd(100) termination.^[8]^ Concerning the extraction of a single Pd atom from the metal surface, this is highly inefficient and would cost energy. Instead, trapping of a diffusing Pd ad‐atom could be much more convenient.[^21^, ^22^, ^23^, ^24^] This is the case when self‐metalation is not assisted by oxygen. Instead, the self‐metalation energy barrier is considerably lowered in many systems upon pre‐oxidation of the metal termination in UHV, including the Pd surface,^[8]^ and as recently reported for the cobalt oxides terminations, where the local substrate structure plays a relevant role.^[10]^ This lowers both the reaction barrier for the extraction of a surface atom and the energy of the final state, since water is produced instead of molecular hydrogen. We thus believe that the oxygen‐induced extraction of a Pd atom is likely in our case, but there is no direct experimental evidence in either sense yet. The experimental data reported in Figure 3 were obtained by means of different techniques (IR–Vis SFG and XPS) in two different experimental setups and at different O_2_ background pressures. The only, single free parameter of the model to fit the experimental data was the activation barrier Δ*E*_1_, since all other parameters were pre‐determined by the experimental conditions. Nevertheless, an excellent agreement between model and experiment was obtained, yielding compatible self‐metalation activation barriers of 0.4 and 0.37 eV for the two experiments. Closer insight is instead necessary to account for all the observed features in the N 1s core level spectra. Indeed, our experimental evidence shows that, starting from the as‐prepared 2H‐TPP/Pd(100) system yielding a single molecular species, namely 2H‐TPP_A_, exposure to oxygen yields the competing conversion of the former into a new 2H‐TPP_B_ species and into two nonequivalent metalated tetrapyrroles (PdTPP_A_ and PdTPP_B_). In Figure 4 a,b we show the evolution of the surface coverage of these species as a function of the oxygen dose (markers), as extracted from the deconvolution of the N 1s core level spectra plotted in Figure 2 b. The concentration of the three new species grows at the expense of the initial 2H‐TPP_A_ population, the coverage of the PdTPP_A_ species reaching a maximum after about 4000 L O_2_. On the basis of literature data,[^8^, ^13^] we associate the PdTPP_A_ species (N 1s at 397.5 eV) to intact Pd tetraphenyl porphyrins. On the contrary, B species (both 2H‐TPP_B_ and PdTPP_B_) are interpreted as porphyrins whose phenyl groups have undergone partial dehydrogenation due to reaction with adsorbed oxygen, that is, O/Pd(100) originating from the O_2_ dissociation and from the formation of the sitting‐atop complex, yielding desorbing water and, indeed, adsorbed O atoms. This can be evinced also from the IR–Vis SFG spectra collected in the low‐energy and in the C−H stretching region. In the former case, an overall decrease of the amplitude of the resonances upon metalation (Figure 1) witnesses indeed that both the macrocycle and the phenyl terminations become flat, lying parallel to the surface, thus interacting more strongly with it. The C−H stretching spectra suggest significant changes in the phenyl orientation as well (Figure S2). This more detailed picture would in principle also account for what was already observed under UHV conditions upon heating, where metalation yields the apparently unexplained full consumption of the pre‐adsorbed oxygen coverage (the full metalation of the 2H‐TPP monolayer needs instead only few % ML of oxygen).^[8]^ Previous results obtained on the Cu(111) termination in UHV conditions further support our interpretation,^[25]^ indicating a role of the metal surface in the H exchange and in the hydrogenation/dehydrogenation reactions of the peripheral molecular terminations. To validate our picture, we implement a second kinetic model (kinetic model 2, see Supporting Information) introducing a second activation barrier (Δ*E*_2_) to describe the phenyl dehydrogenation reaction by surface atomic oxygen, both for metalated and unmetalated porphyrins, yielding the B porphyrin species (orange path in the scheme in Figure 3 c). Here we assume that 2H‐TPP_B_ molecules do not undergo metalation on the timescale/oxygen exposure range covered by the XPS experiment, as evident from the experimental data in Figure 4 a. Continuous, black lines in Figure 4 represent the best fit of the data obtained with the model. The global optimization (all curves were fitted at the same time) of only two free parameters, that is, the activation barriers for metalation and phenyl dehydrogenation, well reproduces the observed trends for all the involved species, yielding Δ*E*_1_=0.37 eV (in perfect agreement with both the simpler model and the IR–Vis SFG data) and Δ*E*_2_=0.91 eV, respectively. A very interesting side result is shown in Figure 4 c, where the atomic oxygen coverage available at the Pd(100) surface is shown as obtained from the model by integration of the differential equations. Interestingly, a maximum coverage of only 0.004 ML is attained, thus explaining why the O 1s core level spectra (Figure S3) do not reveal the presence of adsorbed atomic oxygen, in agreement with the UHV results,^[8]^ indicating complete consumption of surface oxygen upon metalation of the porphyrins. While oxygen is directly involved in the self‐metalation and water production reactions, our data suggest that the TPP monolayer screens the Pd surface from impinging O_2_. In fact, both accumulation of adsorbed oxygen and oxidation of the metal termination are not observed experimentally, thus strongly supporting a direct Eley–Rideal mechanism as implemented in our models.

## Conclusion

In summary, we show that the self‐metalation of tetraphenyl porphyrins can be induced at the Pd(100) surface by gas‐phase oxygen already at room temperature. The reaction is activated, with a barrier of only 0.37–0.4 eV. The best candidate for the rate‐determining step is the formation of the sitting‐atop complex,[^4^, ^6^, ^7^, ^21^, ^25^, ^26^, ^27^] which is typical for porphyrin metalation mechanisms in solution and was proposed for surface metalation mechanisms as well.^[27]^ Within this picture, the pyrrolic hydrogen atoms react via the catalytic mediation of a Pd atom, thus forming molecular hydrogen. In a similar picture the same pyrrolic hydrogen atoms can be transferred instead to an oxygen atom via the catalytic mediation of the Pd atom, thus forming water.[^8^, ^13^] An alternative route, recently proposed on the basis of isotopic exchange investigations, includes a role of the metal surface, accommodating the H atoms after the metalation and before desorption of the product.[^7^, ^25^] However, depending on the surface and on the metal, activation barriers obtained from computational and experimental results span from the almost barrierless porphyrin metalation with Fe in the gas phase, to values as high as 1.3–1.5 eV for the inclusion of Fe or Zn at the Ag(111) surface and for the self‐metalation on Cu(111).[^7^, ^25^, ^27^] Our value of 0.4 eV lies therefore in the lowest part of the range, thus supporting accessibility of this novel metalation route already at room temperature.

## Conflict of interest

The authors declare no conflict of interest.

## Supporting information

As a service to our authors and readers, this journal provides supporting information supplied by the authors. Such materials are peer reviewed and may be re‐organized for online delivery, but are not copy‐edited or typeset. Technical support issues arising from supporting information (other than missing files) should be addressed to the authors.

Supporting Information
