# Supplementary material for: Self‐Metalation of Porphyrins at the Solid–Gas Interface
Source: Angew Chem Int Ed Engl. 2021 Nov 9;60(49):25988–93. doi: 10.1002/anie.202111932 (PMC9299001; doi:10.1002/anie.202111932)
Supplement: Supplementary file 1 — Supporting Information [file ANIE-60-25988-s001.pdf]

Angewandte Chemie
Eine Zeitschrift der Gesellschaft Deutscher Chemiker

Supporting Information

Self-Metalation of Porphyrins at the Solid–Gas Interface

Francesco Armillotta, Enrico D'Incecco, Manuel Corva, Matus Stredansky, Jean-Jacques Gallet, Fabrice Bournel, Andrea Goldoni, Alberto Morgante, Erik Vesselli, and Alberto Verdini**

anie_202111932_sm_miscellaneous_information.pdf

Experimental Section and Methods

Sample preparation. The sample preparation procedure adopted in this work was the same as in the case of the analogous metalation experiments performed in UHV on the oxygen pre-covered termination.[1] For both IR-Vis SFG and NAP-XPS experiments, the Pd(100) surface was cleaned by standard sputtering (Ar^+, 1.5 keV) and annealing cycles (800 K) in UHV, yielding a clean (XPS) and ordered (LEED) termination. 5,10,15,20-Tetraphenyl-21H,23H-porphine (2H-TPP) molecules were evaporated from a hot crucible (575 K), the flux being calibrated by means of a quartz microbalance (SFG experiments) or by measuring the N 1*s* core levels (XPS).

Infrared-Visible Sum-Frequency Generation spectroscopy. IR-Vis sum frequency generation vibronic spectroscopy is a non-linear optical technique.[2,3] Measurements were performed in a dedicated setup.[4] A UHV system with a base pressure of 5×10^{-11} mbar hosting standard surface science preparation and characterization techniques is directly coupled with a high-pressure cell for *in situ* IR-Vis SFG spectroscopy. The reactor is equipped with a gas system to handle the reactants pressure in the 10^{-9}-10^{+2} mbar range. The inlet and outlet of the laser beams are provided by UHV-compatible BaF_2 windows. The Pd(100) disc was mounted on Ta wires, used also for resistive heating. The excitation source (Ekspla, 1064 nm, 30 ps, 50 Hz) delivers a 532 nm (2.33 eV) second harmonic visible beam and tunable IR radiation in the 1000-4500 cm^{-1} range. After normalization to the IR and visible excitation intensities, the SFG spectra were analyzed by least-squares fitting to a parametric, effective expression of the nonlinear second-order susceptibility.[3–5] The expression (reported here below) well reproduces the observed lineshapes, accounting for the resonant IR-Vis vibronic transitions and for the nonresonant background, and describing all the interference terms:

$$\frac{I_{SFG}(\omega_{IR})}{I_{vis}I_{IR}(\omega_{IR})} \propto \left| A_{NRes} + \sum_k \frac{A_k e^{i\Delta\varphi_k}}{\omega_{IR} - \omega_k + i\Gamma_k} \right|^2 \qquad (1)$$

A_{NRes} and A_k account for the amplitudes of the nonresonant and k^{th}-resonant contributions, respectively. $\Delta\varphi_k$ is the phase difference between the kth-resonant and nonresonant signals. ω_k is the energy position of the line and Γ_k its Lorentzian broadening, related to the dephasing rate, which in turn stems from the energy lifetime and from the elastic dephasing of the excited vibronic state.[6] In the manuscript, we plot the normalized IR-Vis SFG signal intensity (dots) together with the best fit (lines) and (color-filled curves) the intensity of each resonance and its interference with the nonresonant background. The latter are calculated with the parameters obtained from the fitting procedure following:

$$\frac{I_{SFG,k}(\omega_{IR})}{I_{vis}I_{IR}(\omega_{IR})} \propto \left| A_{NRes} + \frac{A_k e^{i\Delta\varphi_k}}{\omega_{IR} - \omega_k + i\Gamma_k} \right|^2 \qquad (2)$$

These plots interestingly put in direct evidence the amplitude and the relative phase for each of the resonances. Further details and examples can be found in our previous work.[4,7,8] In the

present study, all spectra were collected in the ppp polarization configuration (SFG-visible-infrared).

Near-Ambient Pressure X-Ray Photoelectron Spectroscopy. The NAP-XPS experiments were performed at the TEMPO beamline of the French national synchrotron radiation facility (SOLEIL, Saint-Aubin) on the setup of the Sorbonne Université. The analyzer of the experimental station is a NAP-XPS Phoibos 150 (Specs GmbH). Further details can be found elsewhere.[9] The N 1*s* core level spectra were collected with a photon energy of 550 eV at an analyzer pass energy of 20 eV.

Quantitative information was obtained by least square fitting of the data after normalization and subtraction of a linear background. For the deconvolution we adopted a Doniach-Šunjic lineshape,[10] convoluted with a Gaussian envelope to account for experimental resolution, inhomogeneity, and phonon broadening. In the case of the N 1*s* contributions from the 2H-TPPs, the metallicity of the surface was not inducing asymmetric line profiles associated with inelastic processes, thus calling for a vanishing asymmetry parameter and letting the photoemission lineshape converge to a Voigt profile. In order to minimize the number of degrees of freedom for the least square fitting analysis, some assumptions have been done. In the specific, for each doublet corresponding to iminic and pyrrolic N atoms contributions, the intensity ratio of the respective components was forced to 1, and the lineshape of both peaks in both doublets was assumed to be the same Voigt. A common lineshape was obtained also for the two singlet peaks, but in this case, the asymmetry was obtained from the fitting procedure.

The time-dependent measurements were performed *in situ*. We observed that the X-ray beam was inducing part of the system evolution, thus affecting the apparent metalation kinetics in agreement with previous observations.[11] We therefore carefully investigated this aspect and, thanks to the motorization of the sample stage, actually performed the measurements by raster-scanning the sample surface. Each spectrum of the series was integrated on a fresh sample spot in a time window (tens of seconds) that was definitely shorter than the evolution induced by the beam (minutes) and almost negligible with respect to the selected time scale of the metalation process (hours). The latter was properly tuned by choosing the oxygen background pressure so to induce a slow evolution of the molecular adlayer, allowing the collection of statistically solid spectroscopic information. Indeed, the nitrogen surface coverage in a full 2H-TPP monolayer is small (of the order of 0.1 ML), thus generating a proportionally small signal, despite the large ionization cross section of the N 1*s* core level at the selected photon energy.

Stability of the H_2TPP monolayer

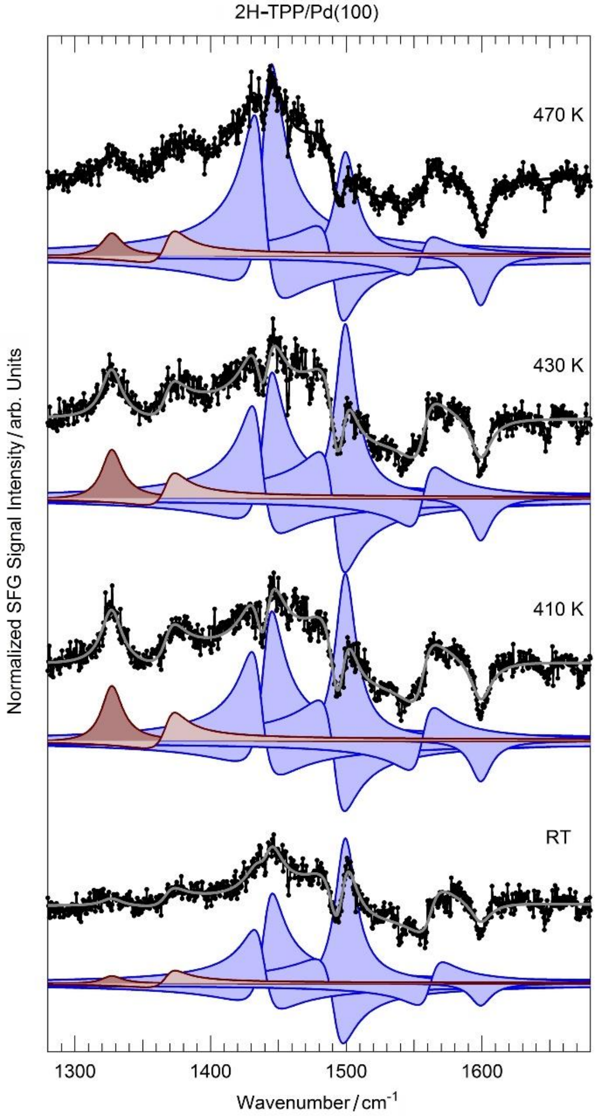

Figure S1. Temperature-dependent IR-Vis SFG spectra of 1 ML of 2H-TPP in UHV, deposited at room temperature on the bare Pd(100) surface. Upon annealing to 410 K we observe an overall increase in the resonances amplitude, suggesting better ordering of the adlayer. However, no significant spectral changes are evident up to 470 K, thus indicating good stability of the molecules and absence of self-metalation fingerprints.

IR-Vis SFG in the C-H stretching region

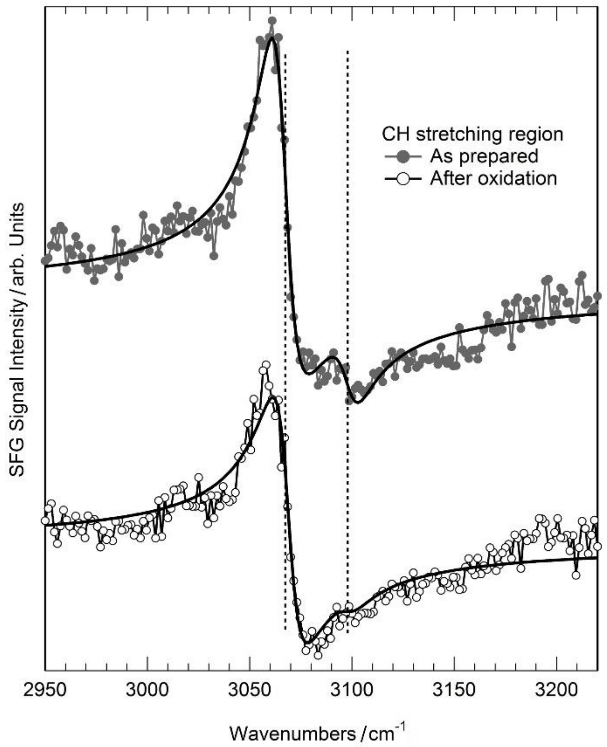

Figure S2. IR-Vis SFG spectra collected in UHV after preparation of 1 ML 2H-TPP/Pd(100) (top) and *in situ* in 1 mbar O_2 (bottom). Two vibronic features are evidenced by the best fitting procedure according to the lineshape described in the Methods section. In the specific, a strong resonance with a dispersive shape ($\varphi = 5° \pm 10°$) is found at 3067 cm^{-1}, while a less intense feature is distinguished at higher energy (3098 cm^{-1}). According to the literature,[12] the two modes can be associated with the in- and out-of-plane combined C-H modes of the peripheral phenyl rings. In oxygen atmosphere (bottom curve), the amplitude of both resonances is diminished, by about 10% and 75 %, respectively. This is compatible with a partial dehydrogenation and a realignment of the phenyl terminations, laying parallel to the surface in the latter case. Mild annealing of the system to 410 K in 1 mbar O_2 (not shown) leads to the complete disappearance of the resonances in this region.

Oxygen coverage and XPS measurements

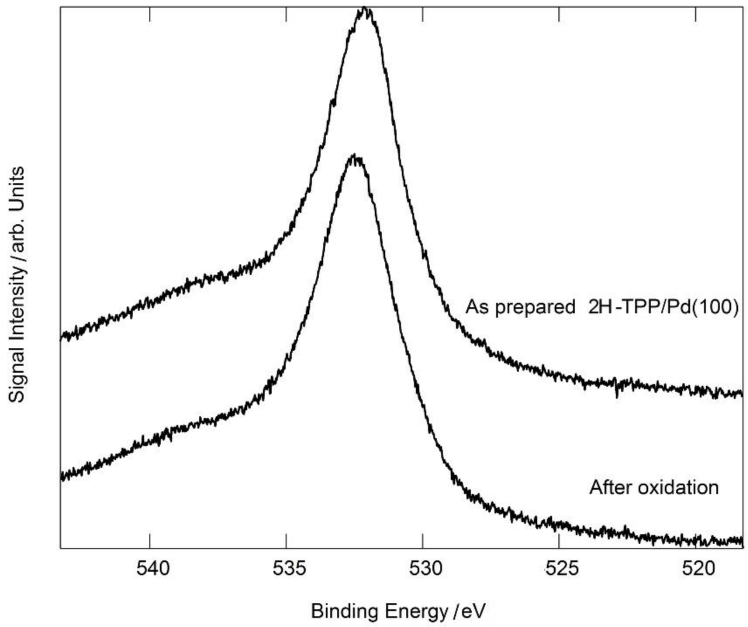

Figure S3. XPS spectra in the Pd $3p_{3/2}$ and O $1s$ region of 1 ML 2H-TPP/Pd(100) collected in UHV before (top) and after (bottom) the oxidation/metalation process. Oxygen adsorbed at the palladium termination, namely O/Pd(100), is expected to contribute spectroscopically at about 530 eV with a sharp and evident peak.[1] Interestingly, only the Pd contribution is observed (photon energy was 700 eV) and no significant spectral modifications can be noticed, apart from a slight lineshape change and binding energy shift of the main peak associated with Pd.

Kinetic model (1)

Within this model, oxygen molecules impinge from the gas phase following the Knudsen flux description. They dissociate at the tetrapyrrole sites, one O atom concurring in the formation of the sitting-atop-complex preceding metalation (activation barrier ΔE_1), a second O atom sticking to the Pd surface underneath. The reaction proceeds at a rate that is proportional to: i) the sticking coefficient s_0 for an activated mechanism (Boltzmann factor); ii) the number/density of unmetalated tetraphenyl porphyrin molecules; iii) the number/density of free sites at the Pd(100) surface to accommodate the oxygen atom originating from O_2 dissociation.

$$\frac{d\theta_{H_2TPP}}{dt} = \frac{ps_0}{\sqrt{2\pi m_{O_2} k_B T}} \left(1 - \theta_{PdTPP_{A+B}}\right)(1 - \theta_O)$$

$$\frac{d\theta_O}{dt} = \frac{ps_0}{\sqrt{2\pi m_{O_2} k_B T}} \left(1 - \theta_{PdTPP_{A+B}}\right)(1 - \theta_O)$$

$$s_0 = e^{-\frac{\Delta E_1}{k_B T}}$$

Kinetic model (2)

With respect to model (1), this model includes also the reaction of O/Pd(100) with hydrogen atoms of the phenyl moieties of both 2H-TPP and PdTPP molecules. This process occurs in parallel with respect to metalation, being secondary to the latter only in the sense that available surface oxygen originates from O_2 reactive adsorption at the center of the tetrapyrroles. Therefore, phenyl rings of both unmetalated (2H-TPP_A) and metalated ($PdTPP_A$) porphyrins can undergo dehydrogenation, yielding 2H-TPP_B and $PdTPP_B$ species, respectively. A barrier ΔE_2 appears in the Boltzmann factor describing activation of the oxidation process, in addition to the ΔE_1 that is associated with the metalation, as in Model (1). Since on the timescale of our spectroscopic data we noticed that dehydrogenated, unmetalated molecules (2H-TPP_B) do not metalate, we did not consider this latter reaction branch. The experiments put in evidence that it is characterized by a higher reaction barrier. In the model we assumed a standard tentative pre-exponential factor of 10^{+13} s^{-1} for the O reaction with H atoms of the phenyl moieties, as previously suggested.[13]

$$\theta_{H_2TPP_A}(t) = 1 - \theta_{H_2TPP_B}(t) - \theta_{PdTPP_A}(t) - \theta_{PdTPP_B}(t)$$

$$\frac{d\theta_{H_2TPP_B}}{dt} = \nu_0 \theta_{H_2TPP_A} \theta_O e^{-\frac{\Delta E_2}{k_B T}}$$

$$\frac{d\theta_{PdTPP_A}}{dt} = \frac{p e^{-\frac{\Delta E_1}{k_B T}}}{\sqrt{2\pi m_{O_2} k_B T}} \theta_{H_2TPP_A}(1 - \theta_O) - \nu_0 \theta_{PdTPP_A} \theta_O e^{-\frac{\Delta E_2}{k_B T}}$$

$$\frac{d\theta_{PdTPP_B}}{dt} = \nu_0 \theta_{PdTPP_A} \theta_O e^{-\frac{\Delta E_2}{k_B T}}$$

$$\frac{d\theta_O}{dt} = \frac{pe^{-\frac{\Delta E_1}{k_B T}}}{\sqrt{2\pi m_{O_2} k_B T}} \theta_{H_2TPP_A}(1-\theta_O) - \nu_0 \theta_{PdTPP_A} \theta_O e^{-\frac{\Delta E_2}{k_B T}} - \nu_0 \theta_{H_2TPP_A} \theta_O e^{-\frac{\Delta E_2}{k_B T}}$$

References

[1] A. Goldoni, P. Shinde, G. L. Montanari, G. Di Santo, M. Caputo, L. Floreano, E. D'Incecco, M. Corva, E. Vesselli, C. A. Pignedoli, et al., *in preparation* **2021**.

[2] Y. R. Shen, *Nature* **1989**, *337*, 519–525.

[3] C. S. Tian, Y. R. Shen, *Surf. Sci. Rep.* **2014**, *69*, 105–131.

[4] M. Corva, Z. Feng, C. Dri, F. Salvador, P. Bertoch, G. Comelli, E. Vesselli, *Phys. Chem. Chem. Phys.* **2016**, *18*, 6763–6772.

[5] M. Hayashi, S. Lin, M. Raschke, Y. Shen, *J. Phys. Chem. A* **2002**, *106*, 2271–2282.

[6] M. Bonn, C. Hess, W. G. Roeterdink, H. Ueba, M. Wolf, *Chem. Phys. Lett.* **2004**, *388*, 269–273.

[7] M. Corva, F. Mohamed, E. Tomsic, M. Rinaldi, C. Cepek, N. Seriani, M. Peressi, E. Vesselli, *J. Phys. Chem. C* **2019**, *123*, 3916–3922.

[8] M. Corva, A. Ferrari, M. Rinaldi, Z. Feng, M. Roiaz, C. Rameshan, G. Rupprechter, R. Costantini, M. Dell'Angela, G. Pastore, et al., *Nat. Commun.* **2018**, *9*, 4703.

[9] A. Naitabdi, A. Boucly, F. Rochet, R. Fagiewicz, G. Olivieri, F. Bournel, R. Benbalagh, F. Sirotti, J.-J. Gallet, *Nanoscale* **2018**, *10*, 6566–6580.

[10] S. Doniach, M. Sunjic, *J. Phys. C Solid State Phys.* **1970**, *3*, 285–291.

[11] C. Bürker, A. Franco-Cañellas, K. Broch, T.-L. Lee, A. Gerlach, F. Schreiber, *J. Phys. Chem. C* **2014**, *118*, 13659–13666.

[12] T. S. Rush, P. M. Kozlowski, C. A. Piffat, R. Kumble, M. Z. Zgierski, T. G. Spiro, *J. Phys. Chem. B* **2000**, *104*, 5020–5034.

[13] M. Röckert, M. Franke, Q. Tariq, D. Lungerich, N. Jux, M. Stark, A. Kaftan, S. Ditze, H. Marbach, M. Laurin, et al., *J. Phys. Chem. C* **2014**, *118*, 26729–26736.
